# High intensity interval training versus moderate intensity continuous training for people with interstitial lung disease: protocol for a randomised controlled trial

**DOI:** 10.1186/s12890-021-01704-2

**Published:** 2021-11-10

**Authors:** Leona M. Dowman, Anthony K. May, Catherine J. Hill, Janet Bondarenko, Lissa Spencer, Norman R. Morris, Jennifer A. Alison, James Walsh, Nicole S. L. Goh, Tamera Corte, Ian Glaspole, Daniel C. Chambers, Christine F. McDonald, Anne E. Holland

**Affiliations:** 1 Respiratory Research @ Alfred, Central Clinical School, Monash University, Melbourne, VIC Australia; 2 Department of Respiratory and Sleep Medicine, Austin Health, Heidelberg, VIC Australia; 3 Department of Physiotherapy, Austin Health, Heidelberg, VIC Australia; 4 Institute for Breathing and Sleep, Melbourne, VIC Australia; 5 Institute for Physical Activity and Nutrition (IPAN), School of Exercise and Nutrition Sciences, Deakin University, Geelong, VIC Australia; 6 Department of Physiotherapy, Alfred Health, Melbourne, VIC Australia; 7 Department of Physiotherapy, Royal Prince Alfred Hospital, Sydney, NSW Australia; 8 School of Health Sciences and Social Work, The Hopkins Centre, Menzies Health Institute Queensland, Griffith University, Gold Coast, QLD Australia; 9 Metro North Hospital and Health Service, The Prince Charles Hospital, Allied Health Research Collaborative, Chermside, QLD Australia; 10 Allied Health Professorial Unit, Sydney Local Health District, Sydney, NSW Australia; 11 Faculty of Medicine and Health Science, Sydney School of Health Sciences, University of Sydney, Sydney, NSW Australia; 12 Department of Respiratory Medicine, Alfred Hospital, Melbourne, VIC Australia; 13 Melbourne Medical School, University of Melbourne, Melbourne, VIC Australia; 14 Department of Respiratory Medicine, Royal Prince Alfred Hospital, Sydney, NSW Australia; 15 Central Clinical School, University of Sydney, Sydney, NSW Australia; 16 NHMRC Centre of Research Excellence in Pulmonary Fibrosis, Sydney, NSW Australia; 17 Central Clinical School, Monash University, Melbourne, VIC Australia; 18 School of Medicine, University of Queensland, Brisbane, QLD Australia; 19 Queensland Lung Transplant Service, The Prince Charles Hospital, Brisbane, QLD Australia

**Keywords:** Interstitial lung diseases, Idiopathic pulmonary fibrosis, Pulmonary Fibrosis, Exercise,, Rehabilitation, High-Intensity interval training, Endurance training

## Abstract

**Background:**

Interstitial lung disease is a debilitating condition associated with significant dyspnoea, fatigue, and poor exercise tolerance. Pulmonary rehabilitation is an effective and key intervention in people with interstitial lung disease. However, despite the best efforts of patients and clinicians, many of those who participate are not achieving clinically meaningful benefits. This assessor-blinded, multi-centre, randomised controlled trial aims to compare the clinical benefits of high intensity interval exercise training versus the standard pulmonary rehabilitation method of continuous training at moderate intensity in people with fibrotic interstitial lung disease.

**Methods:**

Eligible participants will be randomised to either a standard pulmonary rehabilitation group using moderate intensity continuous exercise training or high intensity interval exercise training. Participants in both groups will undertake an 8-week pulmonary rehabilitation program of twice-weekly supervised exercise training including aerobic (cycling) and strengthening exercises. In addition, participants in both groups will be prescribed a home exercise program.

Outcomes will be assessed at baseline, upon completion of the intervention and at six months following the intervention by a blinded assessor. The primary outcome is endurance time on a constant work rate test. Secondary outcomes are functional capacity (6-min walk distance), health-related quality of life (Chronic Respiratory Disease Questionnaire (CRQ), St George’s Respiratory Questionnaire idiopathic pulmonary fibrosis specific version (SGRQ-I), breathlessness (Dyspnoea 12, Modified Medical Research Council Dyspnoea Scale), fatigue (fatigue severity scale), anxiety (Hospital Anxiety and Depression Scale), physical activity level (GeneActiv), skeletal muscle changes (ultrasonography) and completion and adherence to pulmonary rehabilitation.

**Discussion:**

The standard exercise training strategies used in pulmonary rehabilitation may not provide an optimal exercise training stimulus for people with interstitial lung disease. This study will determine whether high intensity interval training can produce equivalent or even superior changes in exercise performance and symptoms. If high intensity interval training proves effective, it will provide an exercise training strategy that can readily be implemented into clinical practice for people with interstitial lung disease.

*Trial registration* ClinicalTrials.gov Registry (NCT03800914). Registered 11 January 2019, https://clinicaltrials.gov/ct2/show/NCT03800914

Australian New Zealand Clinical Trials Registry ACTRN12619000019101. Registered 9 January 2019, https://www.anzctr.org.au/Trial/Registration/TrialReview.aspx?id=376050&isReview=true

## Background

Interstitial lung disease (ILD) encompasses a large group of over 200 debilitating chronic lung conditions that share similar clinical, radiological, histopathological and pathogenetic features and are typically associated with chronic inflammation and fibrosis of lung tissue [1, 2]. Regardless of aetiology, people with ILD often suffer from dyspnoea, fatigue, anxiety, depression, cough, and reduced exercise tolerance. These symptoms are often debilitating and substantially affect health-related quality of life (HRQoL) [3, 4]. Fibrotic ILD (fILD) is a subset of patients where lung pathology is characterised by fibrosis [5, 6]. Some fibrotic ILDs are progressive and associated with a particularly poor prognosis, such as idiopathic pulmonary fibrosis (IPF), the most common and prototypical form of fILD [2]. Other ILD subtypes can develop a progressive fibrosing phenotype and have a similar clinical behaviour to IPF such as idiopathic nonspecific interstitial pneumonia (NSIP), unclassifiable idiopathic interstitial pneumonia (IIP), connective tissue ILD (CTD-ILD) (primarily rheumatoid arthritis (RA)-ILD), chronic hypersensitivity pneumonitis (HP), asbestosis and silicosis [1, 7, 8]. Fibrotic ILD represents a large portion of all ILDs, and people with fILDs typically have more profound dyspnoea, greater physical impairment and disability and a poorer prognosis than those with other types of ILD [1, 5, 7, 8].

There is an accumulation of robust evidence to indicate pulmonary rehabilitation (PR) is an effective and key intervention in ILD. A Cochrane review involving the meta-analysis of 22 randomised controlled trials (RCTs) of PR in ILD [9] found that PR significantly improved functional capacity, HRQoL and dyspnoea. These improvements are comparable in IPF and other ILDs, suggesting PR is effective regardless of aetiology [9]. In addition, current international guidelines support the inclusion of people with ILD in PR [10]. Despite this compelling evidence for the benefits of PR in ILD, the optimal exercise training strategy for people with ILD still needs to be explored. In our earlier RCT assessing the effectiveness of exercise training, utilising the standard PR method of continuous training at moderate intensity, in 142 people with ILD of varying aetiologies [11], less than half of the participants achieved a clinically meaningful improvement in 6-min walk distance (6MWD) following PR [12]. Furthermore, whilst benefits of PR were greatest in individuals who were able to progress the intensity and duration of their exercise training [11], successful progression occurred in only 40% of patients [13], the majority of whom had diagnoses other than IPF [13]. Therefore, current exercise training strategies in PR may not be well suited to people with ILD, particularly those with fILD such as IPF.

Impaired exercise tolerance is a well-known, prominent feature of ILD. In ILD, exercise intolerance is associated with gas exchange impairment and circulatory dysfunction. Probable causes include destruction and remodelling of the pulmonary-capillary vascular bed resulting in reduced diffusing capacity, ventilation perfusion mismatch and pulmonary hypertension. As consequence, people with ILD often present with arterial hypoxemia that worsens during exercise [14–16]. Skeletal muscle dysfunction also contributes to impaired exercise tolerance either due physical deconditioning, increased muscle oxidative stress, arterial hypoxaemia, systemic disease related manifestations and corticosteroids [16–20]. People with fILD tend to experience more severe diffusion limitation, greater exercise induced oxyhaemoglobin desaturation and have more severe pulmonary hypertension compared to those with other ILDs [14, 16, 21, 22]. Therefore, those with fILD often demonstrate a greater limitation to exercise and more profound exertional dyspnoea. These pathophysiological mechanisms of exercise limitation that are accentuated in fILD may limit patients’ ability to adhere to continuous training or to train at a high enough exercise intensity to achieve physiological benefits, therefore resulting in less favourable outcomes.

High intensity interval training (HIIT) may serve as an alternative exercise training regimen to the moderate intensity continuous training (MICT) method typically used in PR. High intensity interval training involves repeated intermittent bursts of high intensity exercise, interspersed by periods of rest or low-intensity exercise [23]. The exercise intervals may be performed at higher intensities than tolerated by continuous training, allowing for an increased training stimulus to peripheral muscles without overloading the cardiorespiratory system [23, 24].The alternating exercise and recovery bouts may also provide intermittent relief from the ventilatory demand of exercise [24], and may reduce the impact of circulatory dysfunction and gas exchange impairment that is often present in those with ILD [14–16]. High intensity interval training has been shown to be effective at improving cardiometabolic health across a range of populations including healthy individuals, people with diabetes, cardiac disease, and heart failure [23, 25, 26].

In chronic obstructive pulmonary disease (COPD), meta-analyses concluded HIIT was equally effective as continuous training in producing beneficial physiological change and improvement in HRQoL [27–29]. Additionally, in some studies HIIT was found to be associated with less leg discomfort [30–32], less dyspnoea [30–32] and greater adherence [33] when compared to continuous training. In ILD, there is little evidence on the benefit of HIIT. One small feasibility study comparing the efficacy of HIIT vs continuous training in ILD of varying aetiology provided preliminary evidence (n = 36) that HIIT is as effective as continuous training [34], although the duration of intervals varied among HIIT participants. Interestingly in their subgroup analysis, HIIT was found to be more effective in patients with IPF, although the sample size was small (approximately n = 12) [34]. This preliminary evidence suggests HIIT may be a more achievable method for people with fILD to attain the recommended exercise training intensity that promotes health-enhancing benefit. A robust, high quality trial is necessary to establish whether HIIT is an effective exercise training strategy in fILD.

The primary aim of this study is to compare the clinical benefits of HIIT vs MICT in people with fILD. We hypothesise that HIIT will deliver clinically significant improvements in exercise endurance (primary outcome), symptoms and HRQoL, which are superior to those achieved with the MICT method typically used in PR.

## Methods

### Design

A randomised, controlled, assessor-blinded trial comparing HIIT to the typical PR method of MICT will be conducted at four metropolitan sites in Australia (Alfred Health and Austin Health in Melbourne, The Prince Charles Hospital in Brisbane, and Royal Prince Alfred Hospital in Sydney). Participants with fILD will be randomized into two groups. Group 1: PR using the standard MICT method and Group 2: PR receiving HIIT. Participants will be followed up for six months following the completion of the allocated 8-week intervention. This six month follow-up period is clinically feasible in fILD without excessive loss to follow up due to transplantation or death [11, 35]. The study protocol (version 5, 19 March 2019) has been approved by the Alfred Hospital Human Research ethics committee with governance approval obtained from all participating sites (Alfred Hospital, Austin Hospital, Royal Prince Alfred Hospital and The Prince Charles Hospital). The study design follows the Standard Protocol Items: Recommendations for Interventional Trials (SPIRIT) checklist (see online supplemental file 1) [36]. The trial is registered at anzctr.org.au (ACTRN12619000019101) and ClinicalTrials.gov **(**NCT03800914).

### Participants

People with fILD who receive care at the participating sites will be invited to participate. Patients will be eligible for inclusion if they are (1) ≥ 18 years of age, (2) able to read and speak English and (3) have a physician-confirmed diagnosis of fILD via high-resolution computed tomography or histology such as IPF, CTD-ILD, HP, fibrotic NSIP, unclassifiable IIP, dust related lung disease or sarcoidosis. People with fILD make up a significant portion of all ILDs and are associated with a significant disease burden (5). Patients with these conditions may be unable to sustain MICT for long durations (15-30 min) possibly due to exertional dyspnoea and exercise induced desaturation [13].

Participants will be excluded if they have (1) a resting oxyhemoglobin saturation (SpO_2_) on room air < 85%; any absolute contraindication to performance of a cardiopulmonary exercise test (CPET) [37]; (2) severe pulmonary hypertension (mean pulmonary arterial pressure > 55 mmHg; World Health Organization (WHO) class IV); (3) attended PR within the last six months; (4) any comorbidities that would preclude exercise training or assessments; (5) a history of syncope on exertion; (6) significant cognitive impairment; (7) admission to an acute care hospital within the last 30 days or (8) if there is anticipated transplantation or death within the study period.

### Recruitment and randomisation

Potential participants will be identified by (1) their treating physician or ILD nurses when attending the respiratory medicine clinic or (2) when they are referred to PR program at the participating sites. Eligible participants will be provided with written and verbal information about the study from a clinician and/or a researcher. Patients will be informed that participation in the study is voluntary and their decision regarding participation will have no effect on their treatment, their relationship with their healthcare team or any other healthcare requirements. All participants will provide written informed consent.

**Fig. 1:**
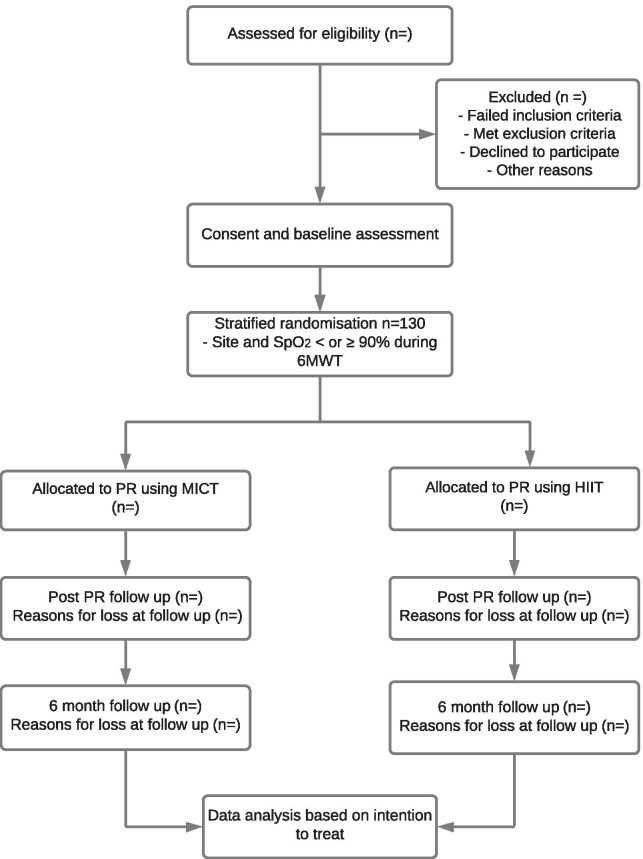
Study Flow. 6MWT, 6-min walk test; HIIT, high intensity interval training; *MICT* moderate intensity continuous training; *PR* pulmonary rehabilitation; *SpO2* oxyhaemoglobin saturation

Following the completion of the baseline assessments, patients will be randomly allocated to receive MICT or HIIT using a computer generated, permuted block randomisation scheme stratified for (i) SpO_2_ < or ≥ 90% during 6-min walk test (6MWT) on room air, to ensure that those who desaturate are evenly distributed between the intervention and control group, and (ii) site of recruitment. Sequence generation will be performed by an individual independent of the research team and the group allocation will be concealed using a secure online database. An independent assessor, blinded to group allocation, will perform the outcome assessments at each timepoint. Given the physical nature of the intervention (exercise training) participants will not be blinded to group allocation. The flow of participants through the study will be reported according to the recommendations from the Consolidated Standards of Reporting Trials statement [38] and is outlined in Fig. 1.

### Interventions

Participants in both groups will undergo a standard 8-week twice-weekly supervised PR at the centre where they were recruited. Each session will consist of lower limb aerobic training plus upper and lower limb resistance training exercises. The intensity (high versus moderate) and the type (continuous versus interval) of aerobic exercise training will differ between the two intervention groups.

#### Moderate intensity continuous training

Participants will perform 30 min of continuous aerobic exercise on a cycle ergometer each session. This can be completed in one or more bouts (e.g., 3 × 10 min, 2 × 15 min, or 1 × 30 min) as tolerated by the participant. The initial intensity of the stationary cycling will be set at the work rate equivalent to 60% of the peak work rate (Wpeak) achieved on the CPET [39, 40]. This approach represents the current standard practice for exercise training in PR programs for chronic lung disease [40–43].

#### High intensity interval training

Participants will perform 36 min of stationary cycling alternating 30-s exercise intervals at 100% of Wpeak achieved during a CPET, with 30 s unloaded pedalling or rest. Similarly, this can be performed in one or more bouts (e.g., 1 × 36 min, 2 × 18 min or 3 × 12 min) as tolerated by the participant. Informed by our pilot study, this interval format attenuated exertional desaturation and was associated with a similar symptom, oxygen consumption (VO_2_) and heart rate response when compared to moderate intensity continuous exercise [44] The volume of exercise, calculated as the product of exercise time and percentage of Wpeak, for HIIT which is 18 min at 100% Wpeak, is work-matched with the MICT volume of exercise of 30 min at 60% peak.

Exercise training and weekly progressions will be standardised across all sites. The intensity of cycle training will be progressed gradually over the 8-week period, as tolerated, based on patient symptoms, towards an overall 20% increase in work rate for both training groups. The resistance program for both groups will comprise at least two upper-body and two lower-body exercises, initially set at a load to achieve 8–12 repetitions for 2 sets of each exercise [41]. All participants will be encouraged to perform three additional unsupervised home-based sessions each week which will be documented in a home diary that is reviewed weekly by the supervising clinician. The home exercise sessions will include walking or cycling dependant on equipment availability and patient preference and strengthening activities utilising equipment readily available in the home environment. Supplemental oxygen will be provided during training if the participant is on long term and/or ambulatory oxygen therapy or if SpO_2_ on room air is less than 85% whilst exercising and is accompanied by symptoms and signs of severe hypoxemia. Supplemental oxygen will be used during home exercise in participants prescribed long term or ambulatory oxygen therapy.

### Outcome measures

Outcome measures will be collected at baseline, upon completion of the 8-week intervention period, and at six months follow up by an assessor who is blinded to the group allocation. At baseline, a symptom limited incremental cycle test (CPET) according to the international standardised protocol [37] will be performed (1) to establish a work rate for the constant work rate test (CWRT) and (2) to set the exercise training intensity for both groups at a percentage of the Wpeak. Data collection will also include age, sex, body mass index, past medical history, use of oxygen therapy, and current pharmacological treatment. Spirometry, completed as per usual care, will be collected at baseline and six months to document disease progression.

### Primary outcome

The primary outcome is change in endurance time measured using the CWRT on a cycle ergometer at 80% of Wpeak achieved on a baseline CPET according to the standardised criteria [37, 45, 46]. All tests will be performed on room air. The CWRT will be terminated before volitional symptom limitation if SpO_2_ < 78%. Endurance time is considered the most responsive outcome for evaluating exercise-related physiological changes following therapeutic interventions in COPD [45–47] and IPF [48].

### Secondary outcomes

Change in functional exercise capacity will be assessed using the 6MWD, obtained from 6MWT. The 6MWD is responsive to change following exercise training in ILD [49]. Two tests will be conducted at each time point, according to international standards [49], to control for the known learning effect with the best distance recorded [49]. Supplemental oxygen will be used during the 6MWTs in those participants already receiving oxygen therapy with flow rates held constant for subsequent tests.

Health related quality of life (HRQoL) will be measured using the St George’s Respiratory Questionnaire IPF specific version (SGRQ-I), and the Chronic Respiratory Disease Questionnaire (CRQ). Both the CRQ and SGRQ-I have been validated in ILD and are responsive to change following exercise training [9, 50].

The Dyspnoea-12 and Modified Medical Research Council (mMRC) dyspnoea scale will be used to assess change in dyspnoea. The Dyspnea-12 assesses breathlessness severity incorporating both the physical and emotional components and has demonstrated reliability in ILD [51–53]. The mMRC is a single five-point scale used to measure the degree of disability that breathlessness poses on day-to-day activities and is a valid measure of dyspnoea and symptom severity in ILD [54–56]. Change in fatigue will be measured using the Fatigue Severity Scale (FSS). The FSS is a valid, sensitive measure in ILD with responsiveness to change following exercise training [57, 58]. Anxiety and Depression will be evaluated using the Hospital Anxiety and Depression Scale (HADS), a validated and widely used tool for assessing psychological distress [59, 60].

Cross-sectional area of the quadriceps will be measured by B-mode ultrasonography to assess changes in muscle size. Ultrasonography is a valid and reliable non-invasive method to measure quadriceps muscle size [61] and it has previously used to measure quadriceps cross-sectional area in COPD and ILD patients [17, 62, 63].

Sedentary behaviour and time spent in physical activity of various intensities (light, moderate, vigorous) will be measured by the GENEActiv actiwatch (ActivInsights Ltd, Kimbolton, Cambridgeshire, United Kingdom), a wrist-worn, waterproof, tri-axial accelerometer with demonstrated validity in measuring physical activity energy expenditure in IPF [64]. Participants will wear the GENEActiv actiwatch continually for 7 days in order to accurately capture all activity intensities [65].

A global rating of change scale will be used to assess the participants’ self-perceived improvement or deterioration in their symptoms and walking ability upon completion of the intervention [66]. The proportion of participants who a) complete the program and b) adhere to the planned exercise protocol will be compared between MICT and HIIT at eight weeks. Attendance of at least 75% of planned sessions will be defined as completion of the intervention [67]. Serious adverse events will be monitored and recorded prospectively during the duration of the study. Adverse events will be defined as progression of fILD (acute respiratory deterioration, development of resting hypoxaemia); acute exacerbation of fILD, musculoskeletal injury, lower limb pain, fainting, dizziness, chest pain, hospitalization; or death. Participants who experience an adverse event will receive all necessary medical care from their local health care team.

### Sample size

A total of 122 participants (61 per group) will be required to detect a clinically important difference between groups for the primary outcome measure of exercise endurance in the CWRT, with 80% power, and at the two-sided 5% level. This is based on the intermediate boundary (150 s) of the mean important difference range for the CWRT (SD = 292 s) [46, 68]. We will also be powered for the secondary outcome of HRQoL measured by the SGRQ-I based on data from our previous RCT of exercise training in ILD [11]. For HRQoL, a total of 114 participants are required to detect a mean difference of 6 points in SGRQ-I total score (SD = 11.3 points). In our previous trial, we had 5% attrition at completion of exercise training and 12% attrition at six months follow up [11]. Therefore, we will randomise a total of 130 participants, split evenly between groups.

### Statistical analysis

Continuous outcome variables will be analysed using linear mixed models, controlling for recruitment site and baseline values as required. All data will be analysed by intention to treat, with inclusion of all available data regardless of whether the intervention is completed. The proportion of participants who completed the program, adhered to the exercise training protocol and had an adverse event will be compared between groups using a chi-squared test. Alpha will be set at 0.05.

### Data integrity, management and monitoring

Hardcopy original data collection forms will be kept in a locked filing cabinet within a locked office. Electronic data will be stored in a purpose-built on-line database (www.adeptrs.com), with encryption and password protection. No identifying information will be stored in the online database. Electronic data for all sites will be accessible by the principal investigator and the trial coordinator. Site specific investigators will only have access to data relating to their individual site. The trial will be monitored by an independent Data Safety and Monitoring Committee (DSMB), chaired by a respiratory physician who is independent of the study team and trial sites. Any serious adverse events will be notified to the overseeing ethics committee (Alfred Health) and the relevant site governance committee, as well as to the DSMB.

## Discussion

Fibrotic ILD is a debilitating condition associated with significant dyspnoea, fatigue, and poor exercise tolerance. Considerable evidence exists that demonstrates PR is an effective intervention that can positively impact these aspects, yet a substantial proportion of participants are not achieving these benefits [12]. Optimising the exercise training approach for people with fILD ensures a greater proportion will achieve the health benefits associated with PR.

Currently the optimal exercise training strategy for people with fILD remains unknown. HIIT however offers promise. The basic premise of HIIT is that individuals are able to exercise at a higher intensity, albeit in short, repeated bouts, providing a greater training stimulus, particularly to the peripheral muscles, without increasing breathlessness or fatigue [23, 24, 28] potentially leading to superior physiological improvements. [28]. In COPD, despite marked heterogeneity in training volume, interval duration and intensity among protocols, HIIT was found to be equally effective to continuous training for improving functional capacity and HRQoL [27, 28, 69] whilst potentially inducing less symptoms of dyspnoea and leg discomfort [31, 32]. In ILD, the evidence for HIIT is limited to one feasibility study with a small sample size and variable intervals among participants [34]. This will be the first RCT to compare the clinical benefits of HIIT vs the commonly used method of MICT over the course of an 8-week PR program in people with fILD. A large sample size with recruitment across four sites, a diverse spectrum of meaningful outcomes that aim to capture endurance and functional exercise capacity, symptoms, muscles changes and daily activity, and a volume matched exercise protocol that is standardized across all the participating sites will ensure an impactful and high quality study.

This study will provide invaluable insight into the benefit of HIIT in fILD. The findings will demonstrate whether HIIT can produce equivalent or even superior improvements in exercise tolerance, symptoms and HRQoL to that of MICT in people with fILD. If HIIT proves successful, it will provide an important, readily available, exercise training strategy that can maximise the outcomes of PR for people with fILD.

### Trial status

Recruitment commenced in July 2019.

## Data Availability

The datasets used and/or analysed during the current study are available from the corresponding author on reasonable request.

## Abbreviations

6MWD: 6-Minute walk distance
6MWT: 6-Minute walk test
CWRT: Constant work rate test
COPD: Chronic obstructive pulmonary disease
CPET: Cardiopulmonary exercise test
CRQ: Chronic respiratory disease questionnaire
CTD-ILD: Connective tissue related ILD
DSMB: Data Safety and Monitoring Committee
FSS: Fatigue severity scale
fILD: Fibrotic interstitial lung disease
HADs: Hospital Anxiety and Depression Scale
HIIT: High intensity interval training
HP: Hypersensitivity pneumonitis
HRQoL: Health-related quality of life
IIP: Unclassifiable idiopathic interstitial pneumonia
ILD: Interstitial lung disease
IPF: Idiopathic pulmonary fibrosis
mMRC: Modified Medical Research Council
MICT: Moderate intensity continuous training
PR: Pulmonary rehabilitation
RA: Rheumatoid arthritis
RCT: Randomised controlled trial
SD: Standardised deviation
SpO_2_: Oxygen saturation
SGRQ-I: St George’s Respiratory Questionnaire idiopathic pulmonary fibrosis specific version
VO_2_: Oxygen consumption
Wpeak: Peak work rate
WHO: World Health Organisation
